# iNR-Drug: Predicting the Interaction of Drugs with Nuclear Receptors in Cellular Networking

**DOI:** 10.3390/ijms15034915

**Published:** 2014-03-19

**Authors:** Yue-Nong Fan, Xuan Xiao, Jian-Liang Min, Kuo-Chen Chou

**Affiliations:** 1 Computer Department, Jing-De-Zhen Ceramic Institute, Jingdezhen 333046, Jiangxi, China; E-Mails: yuenong.f@163.com (Y.-N.F.); minjianliang@126.com (J.-L.M.); 2 Information School, ZheJiang Textile & Fashion College, Ningbo 315211, China; 3 Center of Excellence in Genomic Medicine Research (CEGMR), King Abdulaziz University, Jeddah 21589, Saudi Arabia; E-Mail: kcchou@gordonlifescience.org; 4 Gordon Life Science Institute, 53 South Cottage Road, Belmont, MA 02478, USA

**Keywords:** nuclear receptors (NRs), molecular fingerprints, pseudo amino acid composition, support vector machines (SVMs)

## Abstract

Nuclear receptors (NRs) are closely associated with various major diseases such as cancer, diabetes, inflammatory disease, and osteoporosis. Therefore, NRs have become a frequent target for drug development. During the process of developing drugs against these diseases by targeting NRs, we are often facing a problem: Given a NR and chemical compound, can we identify whether they are really in interaction with each other in a cell? To address this problem, a predictor called “iNR-Drug” was developed. In the predictor, the drug compound concerned was formulated by a 256-D (dimensional) vector derived from its molecular fingerprint, and the NR by a 500-D vector formed by incorporating its sequential evolution information and physicochemical features into the general form of pseudo amino acid composition, and the prediction engine was operated by the SVM (support vector machine) algorithm. Compared with the existing prediction methods in this area, iNR-Drug not only can yield a higher success rate, but is also featured by a user-friendly web-server established at http://www.jci-bioinfo.cn/iNR-Drug/, which is particularly useful for most experimental scientists to obtain their desired data in a timely manner. It is anticipated that the iNR-Drug server may become a useful high throughput tool for both basic research and drug development, and that the current approach may be easily extended to study the interactions of drug with other targets as well.

## 1. Introduction

**Figure 1.**
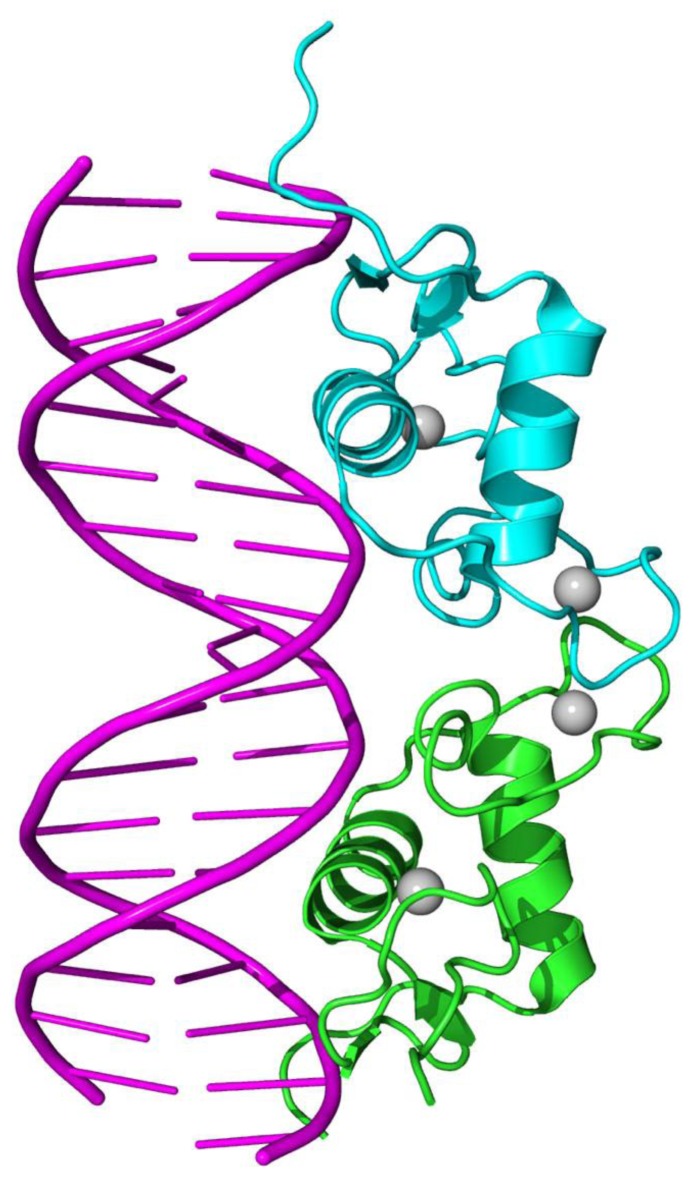
An illustration to show a nuclear receptor binding to DNA.

With the ability to directly bind to DNA (Figure 1) and regulate the expression of adjacent genes, nuclear receptors (NRs) are a class of ligand-inducible transcription factors. They regulate various biological processes, such as homeostasis, differentiation, embryonic development, and organ physiology [1–3]. The NR superfamily has been classified into seven families: NR0 (knirps or DAX like) [4,5]; NR1 (thyroid hormone like), NR2 (HNF4-like), NR3 (estrogen like), NR4 (nerve growth factor IB-like), NR5 (fushi tarazu-F1 like), and NR6 (germ cell nuclear factor like). Since they are involved in almost all aspects of human physiology and are implicated in many major diseases such as cancer, diabetes and osteoporosis, nuclear receptors have become major drug targets [6,7], along with G protein-coupled receptors (GPCRs) [8–17], ion channels [18–20], and kinase proteins [21–24].

Identification of drug-target interactions is one of the most important steps for the new medicine development [25,26]. The method usually adopted in this step is molecular docking simulation [27–43]. However, to make molecular docking study feasible, a reliable 3D (three dimensional) structure of the target protein is the prerequisite condition. Although X-ray crystallography is a powerful tool in determining protein 3D structures, it is time-consuming and expensive. Particularly, not all proteins can be successfully crystallized. For example, membrane proteins are very difficult to crystallize and most of them will not dissolve in normal solvents. Therefore, so far very few membrane protein 3D structures have been determined. Although NMR (Nuclear Magnetic Resonance) is indeed a very powerful tool in determining the 3D structures of membrane proteins as indicated by a series of recent publications (see, e.g., [44–51] and a review article [20]), it is also time-consuming and costly. To acquire the 3D structural information in a timely manner, one has to resort to various structural bioinformatics tools (see, e.g., [37]), particularly the homologous modeling approach as utilized for a series of protein receptors urgently needed during the process of drug development [19,52–57]. Unfortunately, the number of dependable templates for developing high quality 3D structures by means of homology modeling is very limited [37].

To overcome the aforementioned problems, it would be of help to develop a computational method for predicting the interactions of drugs with nuclear receptors in cellular networking based on the sequences information of the latter. The results thus obtained can be used to pre-exclude the compounds identified not in interaction with the nuclear receptors, so as to timely stop wasting time and money on those unpromising compounds [58].

Actually, based on the functional groups and biological features, a powerful method was developed recently [59] for this purpose. However, further development in this regard is definitely needed due to the following reasons. (a) He *et al*. [59] did not provide a publicly accessible web-server for their method, and hence its practical application value is quite limited, particularly for the broad experimental scientists; (b) The prediction quality can be further enhanced by incorporating some key features into the formulation of NR-drug (nuclear receptor and drug) samples via the general form of pseudo amino acid composition [60].

The present study was initiated with an attempt to develop a new method for predicting the interaction of drugs with nuclear receptors by addressing the two points.

As demonstrated by a series of recent publications [10,18,61–70] and summarized in a comprehensive review [60], to establish a really effective statistical predictor for a biomedical system, we need to consider the following steps: (a) select or construct a valid benchmark dataset to train and test the predictor; (b) represent the statistical samples with an effective formulation that can truly reflect their intrinsic correlation with the object to be predicted; (c) introduce or develop a powerful algorithm or engine to operate the prediction; (d) properly perform cross-validation tests to objectively evaluate the anticipated accuracy of the predictor; (e) establish a user-friendly web-server for the predictor that is accessible to the public. Below, let us elaborate how to deal with these steps.

## 2. Results and Discussion

### 2.1. Benchmark Dataset

The data used in the current study were collected from KEGG (Kyoto Encyclopedia of Genes and Genomes) [71] at http://www.kegg.jp/kegg/. KEGG is a database resource for understanding high-level functions and utilities of the biological system, such as the cell, the organism and the ecosystem, from molecular-level information, especially large-scale molecular datasets generated by genome sequencing and other high-throughput experimental technologies. Here, the benchmark dataset 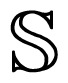 can be formulated as

(1) $$S=S^{+}\cup S^{-}$$

where 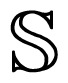
^+^ is the positive subset that consists of the interactive drug-NR pairs only, while 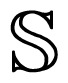
^−^ the negative subset that contains of the non-interactive drug-NR pairs only, and the symbol ∪ represents the union in the set theory. The so-called “interactive” pair here means the pair whose two counterparts are interacting with each other in the drug-target networks as defined in the KEGG database [71]; while the “non-interactive” pair means that its two counterparts are not interacting with each other in the drug-target networks. The positive dataset 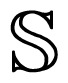
^+^ contains 86 drug-NR pairs, which were taken from He *et al*. [59]. The negative dataset 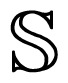
^−^ contains 172 non-interactive drug-NR pairs, which were derived according to the following procedures: (a) separating each of the pairs in 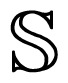
^+^ into single drug and NR; (b) re-coupling each of the single drugs with each of the single NRs into pairs in a way that none of them occurred in 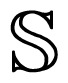
^+^ ; (c) randomly picking the pairs thus formed until reaching the number two times as many as the pairs in 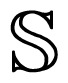
^+^. The 86 interactive drug-NR pairs and 172 non-interactive drug-NR pairs are given in Supplementary Information S1, from which we can see that the 86 + 172 = 258 pairs in the current benchmark dataset 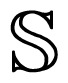 are actually formed by 25 different NRs and 53 different compounds.

### 2.2. Sample Representation

Since each of the samples in the current network system contains a drug (compound) and a NR (protein), the following procedures were taken to represent the drug-NR pair sample.

#### 2.2.1. Use 2D Molecular Fingerprints to Represent Drugs

First, for the drug part in the current benchmark dataset, we can use a 256-D vector to formulate it as given by

(2) $$D={\left[\begin{array}{cccccc} d_{1} & d_{2} & \cdots & d_{i} & \cdots & d_{256} \end{array}\right]}^{T}$$

where **D** represents the vector for a drug compound, and *d_i_* its *i*-th (*i*=1,2, · · ·, 256) component that can be derived by following the “2D molecular fingerprint procedure” as elaborated in [10]. The 53 molecular fingerprint vectors thus obtained for the 53 drugs in 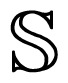 are, respectively, given in Supplementary Information S2.

#### 2.2.2. Use Pseudo Amino Acid Composition to Represent the Nuclear Receptors

The protein sequences of the 25 different NRs in 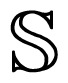 are listed in Supplementary Information S3. Suppose the sequence of a nuclear receptor protein **P** with *L* residues is generally expressed by

(3) $$P={\text{R}}_{1}{\text{R}}_{2}{\text{R}}_{3}{\text{R}}_{4}{\text{R}}_{5}{\text{R}}_{6}{\text{R}}_{7}{\text{R}}_{8}\cdots {\text{R}}_{L}$$

where R_1_ represents the 1st residue of the protein sequence **P**, R_2_ the 2nd residue, and so forth. Now the problem is how to effectively represent the sequence of Equation (3) with a non-sequential or discrete model [72]. This is because all the existing operation engines, such as covariance discriminant (CD) [17,65,73–79], neural network [80–82], support vector machine (SVM) [62–64,83], random forest [84,85], conditional random field [66], nearest neighbor (NN) [86,87]; *K*-nearest neighbor (KNN) [88–90], OET-KNN [91–94], and Fuzzy *K*-nearest neighbor [10,12,18,69,95], can only handle vector but not sequence samples. However, a vector defined in a discrete model may completely lose all the sequence-order information and hence limit the quality of prediction. Facing such a dilemma, can we find an approach to partially incorporate the sequence-order effects?

Actually, one of the most challenging problems in computational biology is how to formulate a biological sequence with a discrete model or a vector, yet still keep considerable sequence order information. To avoid completely losing the sequence-order information for proteins, the pseudo amino acid composition [96,97] or Chou’s PseAAC [98] was proposed. Ever since the concept of PseAAC was proposed in 2001 [96], it has penetrated into almost all the areas of computational proteomics, such as predicting anticancer peptides [99], predicting protein subcellular location [100–106], predicting membrane protein types [107,108], predicting protein submitochondria locations [109–112], predicting GABA(A) receptor proteins [113], predicting enzyme subfamily classes [114], predicting antibacterial peptides [115], predicting supersecondary structure [116], predicting bacterial virulent proteins [117], predicting protein structural class [118], predicting the cofactors of oxidoreductases [119], predicting metalloproteinase family [120], identifying cysteine *S*-nitrosylation sites in proteins [66], identifying bacterial secreted proteins [121], identifying antibacterial peptides [115], identifying allergenic proteins [122], identifying protein quaternary structural attributes [123,124], identifying risk type of human papillomaviruses [125], identifying cyclin proteins [126], identifying GPCRs and their types [15,16], discriminating outer membrane proteins [127], classifying amino acids [128], detecting remote homologous proteins [129], among many others (see a long list of papers cited in the References section of [60]). Moreover, the concept of PseAAC was further extended to represent the feature vectors of nucleotides [65], as well as other biological samples (see, e.g., [130–132]). Because it has been widely and increasingly used, recently two powerful soft-wares, called “PseAAC-Builder” [133] and “propy” [134], were established for generating various special Chou’s pseudo-amino acid compositions, in addition to the web-server “PseAAC” [135] built in 2008.

According to a comprehensive review [60], the general form of PseAAC for a protein sequence **P** is formulated by

(4) $$P={\left[\begin{array}{cccccc} \psi_{1} & \psi_{2} & \cdots & \psi_{u} & \cdots & \psi_{\Omega } \end{array}\right]}^{T}$$

where the subscript Ω is an integer, and its value as well as the components *ψ_u_* (*u* = 1,2, · · ·, Ω) will depend on how to extract the desired information from the amino acid sequence of **P** (*cf.* Equation (3)). Below, let us describe how to extract useful information to define the components of PseAAC for the NR samples concerned.

First, many earlier studies (see, e.g., [136–141]) have indicated that the amino acid composition (AAC) of a protein plays an important role in determining its attributes. The AAC contains 20 components with each representing the occurrence frequency of one of the 20 native amino acids in the protein concerned. Thus, such 20 AAC components were used here to define the first 20 elements in Equation (4); *i.e.*,

(5) $$\psi_{i}=f_{i}^{\left(1\right)}\;\;\;(i=1,2,\cdots ,20)$$

where $f_{i}^{\left(1\right)}$ is the normalized occurrence frequency of the *i*-th type native amino acid in the nuclear receptor concerned. Since AAC did not contain any sequence order information, the following steps were taken to make up this shortcoming.

To avoid completely losing the local or short-range sequence order information, we considered the approach of dipeptide composition. It contained 20 × 20 = 400 components [142]. Such 400 components were used to define the next 400 elements in Equation (4); *i.e.*,

(6) $$\psi_{j+20}=f_{j}^{\left(2\right)}\;\;\;(j=1,2,\cdots ,400)$$

where $f_{j}^{\left(2\right)}$ is the normalized occurrence frequency of the *j*-th dipeptides in the nuclear receptor concerned.

To incorporate the global or long-range sequence order information, let us consider the following approach. According to molecular evolution, all biological sequences have developed starting out from a very limited number of ancestral samples. Driven by various evolutionary forces such as mutation, recombination, gene conversion, genetic drift, and selection, they have undergone many changes including changes of single residues, insertions and deletions of several residues [143], gene doubling, and gene fusion. With the accumulation of these changes over a long period of time, many original similarities between initial and resultant amino acid sequences are gradually faded out, but the corresponding proteins may still share many common attributes [37], such as having basically the same biological function and residing at a same subcellular location [144,145]. To extract the sequential evolution information and use it to define the components of Equation (4), the PSSM (Position Specific Scoring Matrix) was used as described below.

According to Schaffer [146], the sequence evolution information of a nuclear receptor protein **P** with *L* amino acid residues can be expressed by a *L* × 20 matrix, as given by

(7) $$P_{\text{PSSM}}^{\left(0\right)}=\left\lfloor \begin{array}{cccc} E_{1\to 1}^{0} & E_{1\to 2}^{0} & \cdots & E_{1\to 20}^{0}\\ E_{2\to 1}^{0} & E_{2\to 2}^{0} & \cdots & E_{2\to 20}^{0}\\ \vdots & \vdots & \vdots & \vdots \\ E_{L\to 1}^{0} & E_{L\to 2}^{0} & \cdots & E_{L\to 20}^{0} \end{array}\right\rfloor$$

where $E_{i\to j}^{0}$ represents the original score of the *i*-th amino acid residue (*i* = 1, 2,…, *L*) in the nuclear receptor sequence changed to amino acid type *j* (*j* = 1, 2,…, 20) in the process of evolution. Here, the numerical codes 1, 2,…, 20 are used to respectively represent A, C, D, E, F, G, H, I, K, L, M, N, P, Q, R, S, T, V, W, the 20 single-letter codes for the 20 native amino acids. The *L* × 20 scores in Equation (7) were generated by using PSI-BLAST [147] to search the UniProtKB/Swiss-Prot database (The Universal Protein Resource (UniProt); http://www.uniprot.org/) through three iterations with 0.001 as the *E*-value cutoff for multiple sequence alignment against the sequence of the nuclear receptor concerned. In order to make every element in Equation (7) be scaled from their original score ranges into the region of [0, 1], we performed a conversion through the standard sigmoid function to make it become

(8) $$P_{\text{PSSM}}^{\left(0\right)}=\left\lfloor \begin{array}{cccc} E_{1\to 1}^{1} & E_{1\to 2}^{1} & \cdots & E_{1\to 20}^{1}\\ E_{2\to 1}^{1} & E_{2\to 2}^{1} & \cdots & E_{2\to 20}^{1}\\ \vdots & \vdots & \vdots & \vdots \\ E_{L\to 1}^{1} & E_{L\to 2}^{1} & \cdots & E_{L\to 20}^{1} \end{array}\right\rfloor$$

where

(9) $$E_{i\to j}^{1}=\frac{1}{1+{\text{e}}^{-E_{i\to j}^{0}}}\;\;\;(1\leq i\leq L,\;1\leq j\leq 20)$$

Now we extract the useful information from Equation (8) to define the next 20 components of Equation (4) via the following equation

(10) $$\psi_{j+400}=\ell_{j}\;\;\;(j=1,2,\cdots ,20)$$

where

(11) $$\ell_{j}=\frac{1}{L}\times \sum_{k=1}^{L}E_{k\to j}^{1}\;(j=1,2,\cdots ,20)$$

Moreover, we used the grey system model approach as elaborated in [68] to further define the next 60 components of Equation (4); *i.e.*,

(12) $$\psi_{j+400}=\varphi_{j}\;\;\;(j=1,2,\cdots ,60)$$

where

(13) $$\{\begin{array}{l} \varphi_{3j-2}=w_{1}f_{j}^{\left(1\right)}a_{1}^{j}\\ \varphi_{3j-1}=w_{1}f_{j}^{\left(1\right)}a_{2}^{j}\\ \varphi_{3j}=w_{3}f_{j}^{\left(1\right)}b^{j} \end{array}\;\;\;(j=1,2,\cdots ,20)$$

In the above equation, *w*_1_, *w*_2_, and *w*_3_ are weight factors, which were all set to 1 in the current study; $f_{j}^{\left(1\right)}$ has the same meaning as in Equation (5); $a_{1}^{j},a_{2}^{j}$, and *b^j^* are given by

(14) $$\left[\begin{array}{c} a_{1}^{j}\\ a_{2}^{j}\\ b^{j} \end{array}\right]={\left(B_{j}^{T}B_{j}\right)}^{-1}B_{j}^{T}U_{j}\;\;\;(j=1,2,\cdots ,20)$$

where

(15) $$B_{j}=\left[\begin{array}{ccc} -E_{2\to j}^{1} & -\left(E_{1\to j}^{1}+0.5E_{2\to j}^{1}\right) & 1\\ -E_{3\to j}^{1} & -\left(\sum_{i=1}^{2}E_{i\to j}^{1}+0.5E_{3\to j}^{1}\right) & 1\\ \vdots & \vdots & \vdots \\ -E_{L\to j}^{1} & -\left(\sum_{i=1}^{L-1}E_{i\to j}^{1}+0.5E_{L\to j}^{1}\right) & 1 \end{array}\right]$$

and

(16) $$U_{j}=\left[\begin{array}{c} E_{2\to j}^{1}-E_{1\to j}^{1}\\ E_{3\to j}^{1}-E_{2\to j}^{1}\\ \vdots \\ E_{L\to j}^{1}-E_{L-1\to j}^{1} \end{array}\right]$$

Combining Equations (5), (6), (10) and (12), we found that the total number of the components obtained via the current approach for the PseAAC of Equation (4) is

(17) Ω=20+400+20+60=500

and each of the 500 components is given by

(18) $$\psi_{u}=\{\begin{array}{ll} f_{u}^{\left(1\right)} & \text{if }1\leq u\leq 20\\ f_{u}^{\left(2\right)} & \text{if }21\leq u\leq 420\\ \ell_{u} & \text{if }421\leq u\leq 440\\ \varphi_{u} & \text{if }441\leq u\leq 500 \end{array}$$

#### 2.2.3. Formulate the Pair of Drugs with Nuclear Receptor

Since the elements in Equations (2) and (4) are well defined, we can now formulate the drug-NR pair by combining the two equations as given by

(19) $$G=D⊕P=\left[\begin{array}{cccccccc} d_{1} & d_{2} & \cdots & d_{256} & \psi_{1} & \psi_{2} & \cdots & \psi_{500} \end{array}\right]$$

where **G** represents the drug-NR pair, ⊕ the orthogonal sum, and the 256 + 500 = 756 components are defined by Equations (2) and (18).

For the sake of convenience, let us use *x_i_* (*i* =1, 2, · · ·, 756) to represent the 756 components in Equation (19); *i.e.*,

(20) $$G={\left[\begin{array}{cccccc} x_{1} & x_{2} & \cdots & x_{i} & \cdots & x_{756} \end{array}\right]}^{T}$$

To optimize the prediction quality with a time-saving approach, similar to the treatment [148–150], let us convert Equation (20) to

(21) $$G={\left[\begin{array}{cccccc} y_{1} & y_{2} & \cdots & y_{i} & \cdots & y_{756} \end{array}\right]}^{T}$$

where

(22) $$y_{i}=\frac{x_{i}-\langle x_{i}\rangle }{\text{SD}(x)}$$

where the symbol 〈 〉 means taking the average of the quantity therein, and SD means the corresponding standard derivation.

#### 2.2.4. Operation Engine or Algorithm

In this study, the SVM (support vector machine) was used as the operation engine. SVM has been widely used in the realm of bioinformatics (see, e.g., [62–64,151–154]). The basic idea of SVM is to transform the data into a high dimensional feature space, and then determine the optimal separating hyperplane using a kernel function. For a brief formulation of SVM and how it works, see the papers [155,156]; for more details about SVM, see a monograph [157].

In this study, the LIBSVM package [158] was used as an implementation of SVM, which can be downloaded from http://www.csie.ntu.edu.tw/~cjlin/libsvm/, the popular radial basis function (RBF) was taken as the kernel function. For the current SVM classifier, there were two uncertain parameters: penalty parameter *C* and kernel parameter *γ*. The method of how to determine the two parameters will be given later.

**Figure 2.**
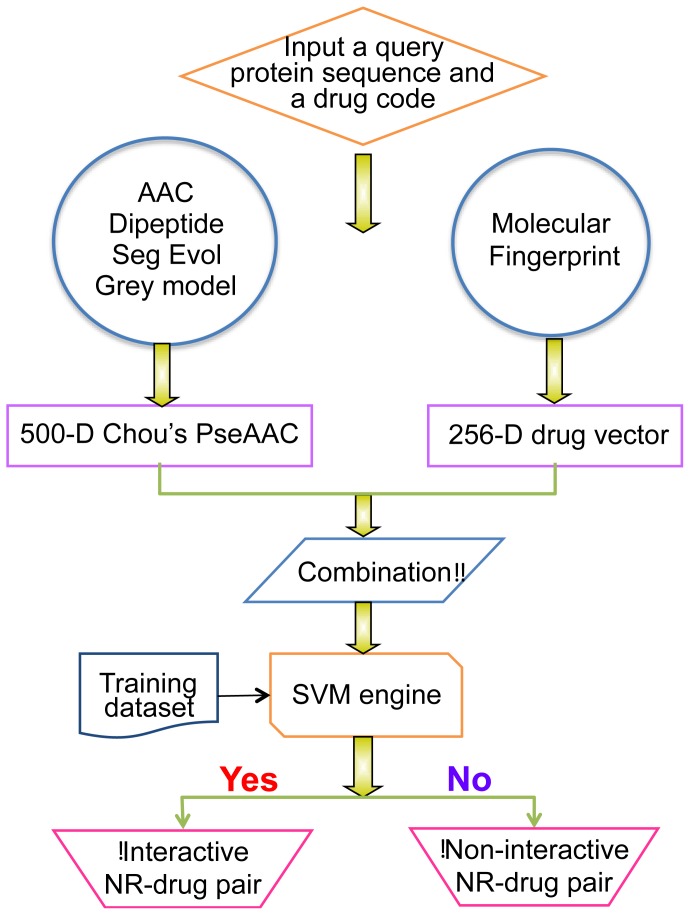
A flowchart to show the operation process of the iNR-Drug predictor.

The predictor obtained via the aforementioned procedure is called iNR-Drug, where “*i*” means identify, and “NR-Drug” means the interaction between nuclear receptor and drug compound. To provide an intuitive overall picture, a flowchart is provided in Figure 2 to show the process of how the predictor works in identifying the interactions between nuclear receptors and drug compounds.

## 3. Experimental Section

### 3.1. Metrics for Measuring Prediction Quality

To provide a more intuitive and easier-to-understand method to measure the prediction quality, the following set of metrics based on the formulation used by Chou [159–161] in predicting signal peptides was adopted. According to Chou’s formulation, the sensitivity, specificity, overall accuracy, and Matthew’s correlation coefficient can be respectively expressed as [62,65–67]

(23) $$\{\begin{array}{l} \text{Sn}=1-\frac{N_{-}^{+}}{N^{+}}\\ \text{Sp}=1-\frac{N_{+}^{-}}{N^{+}}\\ \text{Acc}=1-\frac{N_{-}^{+}+N_{+}^{-}}{N^{+}+N^{-}}\\ \text{MCC}=\frac{1-\left(\frac{N_{-}^{+}+N_{+}^{-}}{N^{+}+N^{-}}\right)}{\sqrt{\left(1-\frac{N_{+}^{-}-N_{-}^{+}}{N^{+}}\right)\left(1+\frac{N_{-}^{+}-N_{+}^{-}}{N^{-}}\right)}} \end{array}$$

where *N*^+^ is the total number of the interactive NR-drug pairs investigated while $N_{-}^{+}$ the number of the interactive NR-drug pairs incorrectly predicted as the non-interactive NR-drug pairs; *N*^−^ the total number of the non-interactive NR-drug pairs investigated while $N_{+}^{-}$ the number of the non-interactive NR-drug pairs incorrectly predicted as the interactive NR-drug pairs.

According to Equation (23) we can easily see the following. When $N_{-}^{+}=0$ meaning none of the interactive NR-drug pairs was mispredicted to be a non-interactive NR-drug pair, we have the sensitivity Sn = 1; while $N_{-}^{+}=N^{+}$ meaning that all the interactive NR-drug pairs were mispredicted to be the non-interactive NR-drug pairs, we have the sensitivity Sn = 0. Likewise, when $N_{+}^{-}=0$ meaning none of the non-interactive NR-drug pairs was mispredicted, we have the specificity Sp = 1; while $N_{+}^{-}=N^{-}$ meaning all the non-interactive NR-drug pairs were incorrectly predicted as interactive NR-drug pairs, we have the specificity Sp = 0. When $N_{-}^{+}=N_{+}^{-}=0$ meaning that none of the interactive NR-drug pairs in the dataset 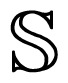
^+^ and none of the non-interactive NR-drug pairs in 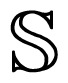
^−^ was incorrectly predicted, we have the overall accuracy Acc = 1; while $N_{-}^{+}=N^{+}$ and $N_{+}^{-}=N^{-}$ meaning that all the interactive NR-drug pairs in the dataset 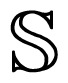
^+^ and all the non-interactive NR-drug pairs in 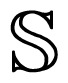
^−^ were mispredicted, we have the overall accuracy Acc = 0. The Matthews correlation coefficient MCC is usually used for measuring the quality of binary (two-class) classifications. When $N_{-}^{+}=N_{+}^{-}=0$ meaning that none of the interactive NR-drug pairs in the dataset 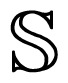
^+^ and none of the non-interactive NR-drug pairs in 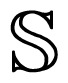
^−^ was mispredicted, we have MCC = 1; when $N_{-}^{+}=N^{+}/2$ and $N_{+}^{-}=N^{-}/2$ we have MCC = 0 meaning no better than random prediction; when $N_{-}^{+}=N^{+}$ and $N_{+}^{-}=N^{-}$ we have MCC = 0 meaning total disagreement between prediction and observation. As we can see from the above discussion, it is much more intuitive and easier to understand when using Equation (23) to examine a predictor for its four metrics, particularly for its Mathew’s correlation coefficient. It is instructive to point out that the metrics as defined in Equation (23) are valid for single label systems; for multi-label systems, a set of more complicated metrics should be used as given in [162].

### 3.2. Jackknife Test Approach

How to properly test a predictor for its anticipated success rates is very important for its development as well as its potential application value. Generally speaking, the following three cross-validation methods are often used to examine the quality of a predictor and its effectiveness in practical application: independent dataset test, subsampling or *K*-fold (such as five-fold, seven-fold, or 10-fold) crossover test and jackknife test [163]. However, as elaborated by a penetrating analysis in [164], considerable arbitrariness exists in the independent dataset test. Also, as demonstrated in [165], the subsampling (or *K*-fold crossover validation) test cannot avoid arbitrariness either. Only the jackknife test is the least arbitrary that can always yield a unique result for a given benchmark dataset [73,74,156,166–168]. Therefore, the jackknife test has been widely recognized and increasingly utilized by investigators to examine the quality of various predictors (see, e.g., [14,15,68,99,106,107,124,169,170]). Accordingly, in this study the jackknife test was also adopted to evaluate the accuracy of the current predictor.

**Figure 3.**
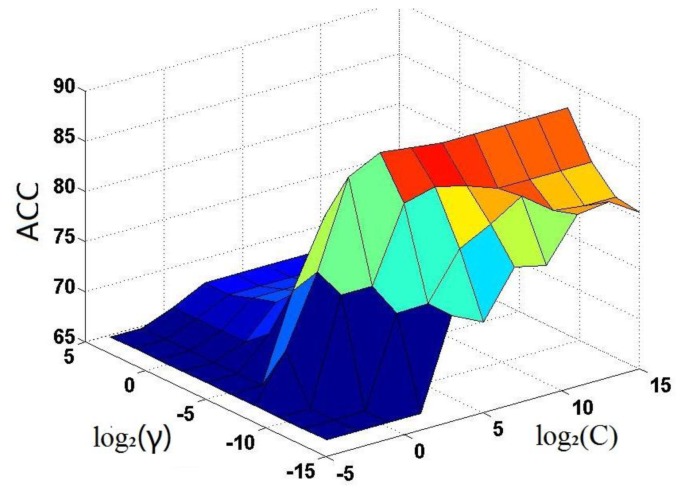
A 3-D graph showing how to optimize the two parameters *γ* and *C* in SVM via the jackknife success rates.

As mentioned above, the SVM operation engine contains two uncertain parameters *C* and *γ*. To find their optimal values, a 2-D grid search was conducted by the jackknife test on the benchmark dataset 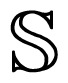. The results thus obtained are shown in Figure 3, from which it can be seen that the iNR-Drug predictor reaches its optimal status when *C* = 2^3^ and *γ* = 2^−9^. The corresponding rates for the four metrics (*cf.* Equation (23)) are given in Table 1, where for facilitating comparison, the overall accuracy Acc reported by He *et al*. [59] on the same benchmark dataset is also given although no results were reported by them for Sn, Sp and MCC. It can be observed from the table that the overall accuracy obtained by iNR-Drug is remarkably higher that of He *et al*. [59], and that the rates achieved by iNR-Drug for the other three metrics are also quite higher. These facts indicate that the current predictor not only can yield higher overall prediction accuracy but also is quite stable with low false prediction rates.

### 3.3. Independent Dataset Test

As mentioned above (Section 3.2), the jackknife test is the most objective method for examining the quality of a predictor. However, as a demonstration to show how to practically use the current predictor, we took 41 NR-drug pairs from the study by Yamanishi *et al*. [171] that had been confirmed by experiments as interactive pairs. For such an independent dataset, 34 were correctly identified by iNR-Drug as interactive pairs, *i.e.*, *i.e.*, Sn=34/41=82.92%, which is quite consistent with the rate of 79.07% achieved by the predictor on the benchmark dataset 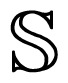 via the jackknife test as reported in Table 1.

## 4. Conclusions

It is anticipated that the iNR-Drug predictor developed in this paper may become a useful high throughput tool for both basic research and drug development, and that the current approach may be easily extended to study the interactions of drug with other targets as well. Since user-friendly and publicly accessible web-servers represent the future direction for developing practically more useful predictors [98,172], a publicly accessible web-server for iNR-Drug was established.

For the convenience of the vast majority of biologists and pharmaceutical scientists, here let us provide a step-by-step guide to show how the users can easily get the desired result by using iNR-Drug web-server without the need to follow the complicated mathematical equations presented in this paper for the process of developing the predictor and its integrity.

**Figure 4.**
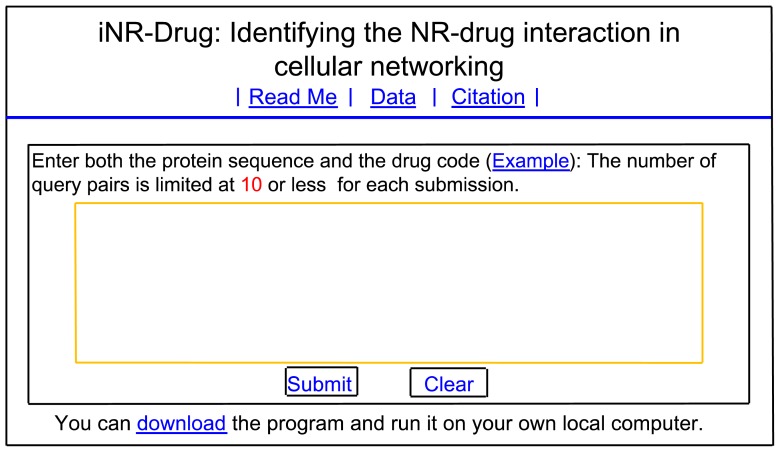
A semi-screenshot to show the top page of the iNR-Drug web-server. Its website address is at http://www.jci-bioinfo.cn/iNR-Drug.

Step 1. Open the web server at the site http://www.jci-bioinfo.cn/iNR-Drug/ and you will see the top page of the predictor on your computer screen, as shown in Figure 4. Click on the Read Me button to see a brief introduction about iNR-Drug predictor and the caveat when using it.

Step 2. Either type or copy/paste the query NR-drug pairs into the input box at the center of Figure 4. Each query pair consists of two parts: one is for the nuclear receptor sequence, and the other for the drug. The NR sequence should be in FASTA format, while the drug in the KEGG code beginning with the symbol #. Examples for the query pairs input and the corresponding output can be seen by clicking on the Example button right above the input box.

Step 3. Click on the Submit button to see the predicted result. For example, if you use the three query pairs in the Example window as the input, after clicking the Submit button, you will see on your screen that the “hsa:2099” NR and the “D00066” drug are an interactive pair, and that the “hsa:2908” NR and the “D00088” drug are also an interactive pair, but that the “hsa:5468” NR and the “D00279” drug are not an interactive pair. All these results are fully consistent with the experimental observations. It takes about 3 minutes before each of these results is shown on the screen; of course, the more query pairs there is, the more time that is usually needed.

Step 4. Click on the Citation button to find the relevant paper that documents the detailed development and algorithm of iNR-Durg.

Step 5. Click on the Data button to download the benchmark dataset used to train and test the iNR-Durg predictor.

Step 6. The program code is also available by clicking the button download on the lower panel of Figure 4.

## Supplementary Information



## Figures and Tables

**Table 1.** The jackknife success rates obtained iNR-Drug in identifying the interactive NR-drug pairs and non-interactive NR-drug pairs for the benchmark dataset 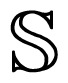 (*cf.* Supplementary Information S1).

Metrics used for measuring prediction quality (*cf.* Equation (23))	iNR-Drug a	Method by He *et al*. b
Sn	$\frac{68}{86}=79.07\%$	N/A
Sp	$\frac{162}{172}=94.19\%$	N/A
Acc	$\frac{230}{258}=89.15\%$	85.66%
MCC	75.19%	N/A

a The parameters used: *C*= 2^3^ and γ= 2^−9^ for the SVM operation engine;

b See [59].
