# Supplementary material for: iNR-Drug: Predicting the Interaction of Drugs with Nuclear Receptors in Cellular Networking
Source: Int J Mol Sci. 2014 Mar 19;15(3):4915–37. doi: 10.3390/ijms15034915 (PMC3975431; doi:10.3390/ijms15034915)
Supplement: Supplementary file 1 [file ijms-15-04915-s001.pdf]

Supplementary Information

Supplementary Information S1. The benchmark dataset contains 258 NR-drug pair samples, of which 86 are interactive and 172 non-interactive. The codes listed here were from the KEGG database at http://www.kegg.jp/kegg/ (KEGG: Kyoto Encyclopedia of Genes and Genomes, Kanehisa Laboratories, Japan).

Pair attribute	Target NRs gene code in KEGG	Drug code
Interactive	hsa:2099	D00066
Interactive	hsa:2099	D00067
Interactive	hsa:2099	D00105
Interactive	hsa:2099	D00312
Interactive	hsa:2099	D00327
Interactive	hsa:2099	D00554
Interactive	hsa:2099	D00577
Interactive	hsa:2099	D00898
Interactive	hsa:2099	D00950
Interactive	hsa:2099	D00954
Interactive	hsa:2099	D00962
Interactive	hsa:2099	D01161
Interactive	hsa:2099	D01294
Interactive	hsa:2099	D02217
Interactive	hsa:2099	D02367
Interactive	hsa:2099	D04066
Interactive	hsa:190	D00094
Interactive	hsa:2100	D00105
Interactive	hsa:2100	D00577
Interactive	hsa:2100	D01161
Interactive	hsa:2100	D02217
Interactive	hsa:2100	D04066
Interactive	hsa:2101	D00577
Interactive	hsa:2103	D00577
Interactive	hsa:2104	D00577
Interactive	hsa:2908	D00088
Interactive	hsa:2908	D00246
Interactive	hsa:2908	D00585
Interactive	hsa:2908	D01387
Interactive	hsa:2908	D01689
Interactive	hsa:3174	D05341
Interactive	hsa:367	D00075
Interactive	hsa:367	D00327
Interactive	hsa:367	D00462
Interactive	hsa:367	D00585
Interactive	hsa:367	D00586
Interactive	hsa:367	D00956
Interactive	hsa:367	D00961
Interactive	hsa:367	D00965
Interactive	hsa:4306	D00443

Supplementary Information S1. *Cont.*

Pair attribute	Target NRs gene code in KEGG	Drug code
Interactive	hsa:4306	D00585
Interactive	hsa:4306	D01115
Interactive	hsa:5241	D00066
Interactive	hsa:5241	D00182
Interactive	hsa:5241	D00585
Interactive	hsa:5241	D00690
Interactive	hsa:5241	D00950
Interactive	hsa:5241	D00951
Interactive	hsa:5241	D00954
Interactive	hsa:5241	D01217
Interactive	hsa:5241	D01294
Interactive	hsa:5241	D02367
Interactive	hsa:5465	D00279
Interactive	hsa:5465	D00565
Interactive	hsa:5468	D00596
Interactive	hsa:5468	D00627
Interactive	hsa:5914	D00094
Interactive	hsa:5914	D00316
Interactive	hsa:5914	D00348
Interactive	hsa:5914	D01132
Interactive	hsa:5915	D00094
Interactive	hsa:5915	D00316
Interactive	hsa:5915	D01132
Interactive	hsa:5916	D00094
Interactive	hsa:5916	D00316
Interactive	hsa:5916	D01132
Interactive	hsa:6095	D00040
Interactive	hsa:6095	D01441
Interactive	hsa:6096	D00094
Interactive	hsa:6096	D01132
Interactive	hsa:6097	D00094
Interactive	hsa:6256	D00094
Interactive	hsa:6256	D00316
Interactive	hsa:6256	D01132
Interactive	hsa:6257	D00094
Interactive	hsa:6257	D00316
Interactive	hsa:6257	D01132
Interactive	hsa:7421	D00187
Interactive	hsa:7421	D00188
Interactive	hsa:7421	D00299
Interactive	hsa:7421	D00930
Interactive	hsa:8856	D00143
Interactive	hsa:8856	D00211
Interactive	hsa:8856	D00730
Interactive	hsa:9970	D00506
Interactive	hsa:9971	D00163

Supplementary Information S1. *Cont.*

Pair attribute	Target NRs gene code in KEGG	Drug code
Non-interactive	hsa:190	D00312
Non-interactive	hsa:190	D00961
Non-interactive	hsa:190	D01689
Non-interactive	hsa:2101	D00040
Non-interactive	hsa:2103	D00690
Non-interactive	hsa:2104	D00316
Non-interactive	hsa:2908	D01132
Non-interactive	hsa:5241	D00596
Non-interactive	hsa:5241	D02217
Non-interactive	hsa:5465	D00506
Non-interactive	hsa:5465	D00961
Non-interactive	hsa:5468	D00279
Non-interactive	hsa:5468	D00348
Non-interactive	hsa:5468	D00954
Non-interactive	hsa:5915	D00279
Non-interactive	hsa:5916	D00965
Non-interactive	hsa:6095	D00961
Non-interactive	hsa:6096	D00182
Non-interactive	hsa:6096	D00327
Non-interactive	hsa:6256	D00312
Non-interactive	hsa:6256	D00565
Non-interactive	hsa:7421	D00443
Non-interactive	hsa:8856	D02217
Non-interactive	hsa:9970	D00182
Non-interactive	hsa:9970	D00961
Non-interactive	hsa:9971	D00565
Non-interactive	hsa:6095	D00585
Non-interactive	hsa:5468	D00730
Non-interactive	hsa:3174	D00690
Non-interactive	hsa:2100	D00565
Non-interactive	hsa:4306	D00965
Non-interactive	hsa:7421	D00961
Non-interactive	hsa:190	D00327
Non-interactive	hsa:9970	D00094
Non-interactive	hsa:5468	D00898
Non-interactive	hsa:5241	D01132
Non-interactive	hsa:6096	D00188
Non-interactive	hsa:5241	D00211
Non-interactive	hsa:190	D00075
Non-interactive	hsa:9970	D01294
Non-interactive	hsa:9971	D00506
Non-interactive	hsa:9971	D01689
Non-interactive	hsa:9971	D00965
Non-interactive	hsa:2104	D00965

Supplementary Information S1. *Cont.*

Pair attribute	Target NRs gene code in KEGG	Drug code
Non-interactive	hsa:6096	D00627
Non-interactive	hsa:6097	D00965
Non-interactive	hsa:4306	D00506
Non-interactive	hsa:3174	D01387
Non-interactive	hsa:6097	D00565
Non-interactive	hsa:2101	D00299
Non-interactive	hsa:5468	D00565
Non-interactive	hsa:190	D00627
Non-interactive	hsa:2101	D00965
Non-interactive	hsa:8856	D00443
Non-interactive	hsa:5915	D00246
Non-interactive	hsa:5468	D00327
Non-interactive	hsa:8856	D00577
Non-interactive	hsa:9970	D00586
Non-interactive	hsa:5916	D00961
Non-interactive	hsa:190	D00950
Non-interactive	hsa:8856	D00040
Non-interactive	hsa:7421	D00898
Non-interactive	hsa:6096	D00163
Non-interactive	hsa:6097	D00690
Non-interactive	hsa:190	D02217
Non-interactive	hsa:9970	D01441
Non-interactive	hsa:9970	D00565
Non-interactive	hsa:2908	D00627
Non-interactive	hsa:9971	D01161
Non-interactive	hsa:367	D02217
Non-interactive	hsa:6097	D01294
Non-interactive	hsa:5241	D00965
Non-interactive	hsa:5465	D00312
Non-interactive	hsa:6095	D00211
Non-interactive	hsa:2101	D00627
Non-interactive	hsa:6095	D00596
Non-interactive	hsa:4306	D04066
Non-interactive	hsa:8856	D00187
Non-interactive	hsa:6096	D00930
Non-interactive	hsa:4306	D00279
Non-interactive	hsa:9970	D00554
Non-interactive	hsa:7421	D00690
Non-interactive	hsa:4306	D01161
Non-interactive	hsa:5465	D04066
Non-interactive	hsa:190	D00187
Non-interactive	hsa:2104	D01294
Non-interactive	hsa:190	D00067
Non-interactive	hsa:190	D00088
Non-interactive	hsa:2100	D00211
Non-interactive	hsa:5465	D00627

Supplementary Information S1. *Cont.*

Pair attribute	Target NRs gene code in KEGG	Drug code
Non-interactive	hsa:5468	D00040
Non-interactive	hsa:6257	D00565
Non-interactive	hsa:7421	D00316
Non-interactive	hsa:9970	D00965
Non-interactive	hsa:190	D01441
Non-interactive	hsa:2100	D01689
Non-interactive	hsa:2101	D02217
Non-interactive	hsa:3174	D04066
Non-interactive	hsa:5465	D00246
Non-interactive	hsa:5468	D00586
Non-interactive	hsa:5916	D01441
Non-interactive	hsa:6095	D02217
Non-interactive	hsa:6096	D00067
Non-interactive	hsa:6096	D00506
Non-interactive	hsa:8856	D00279
Non-interactive	hsa:8856	D00462
Non-interactive	hsa:9970	D00040
Non-interactive	hsa:9970	D00163
Non-interactive	hsa:9971	D00627
Non-interactive	hsa:5241	D00961
Non-interactive	hsa:6256	D00961
Non-interactive	hsa:5465	D01115
Non-interactive	hsa:6257	D00443
Non-interactive	hsa:4306	D00690
Non-interactive	hsa:2100	D00596
Non-interactive	hsa:8856	D00299
Non-interactive	hsa:2101	D00094
Non-interactive	hsa:5915	D01441
Non-interactive	hsa:2101	D00951
Non-interactive	hsa:2101	D01217
Non-interactive	hsa:6097	D00961
Non-interactive	hsa:190	D00956
Non-interactive	hsa:5468	D01115
Non-interactive	hsa:2103	D01387
Non-interactive	hsa:6257	D00965
Non-interactive	hsa:6097	D01441
Non-interactive	hsa:6096	D01161
Non-interactive	hsa:6096	D00143
Non-interactive	hsa:6095	D00565
Non-interactive	hsa:7421	D00211
Non-interactive	hsa:9971	D04066
Non-interactive	hsa:9971	D00182
Non-interactive	hsa:2908	D00586
Non-interactive	hsa:5915	D01161
Non-interactive	hsa:2103	D00730

Supplementary Information S1. *Cont.*

Pair attribute	Target NRs gene code in KEGG	Drug code
Non-interactive	hsa:8856	D00585
Non-interactive	hsa:6097	D00187
Non-interactive	hsa:5468	D00182
Non-interactive	hsa:3174	D00187
Non-interactive	hsa:8856	D04066
Non-interactive	hsa:2908	D00554
Non-interactive	hsa:2101	D01161
Non-interactive	hsa:2100	D00143
Non-interactive	hsa:2103	D00961
Non-interactive	hsa:6257	D00586
Non-interactive	hsa:190	D00316
Non-interactive	hsa:5465	D01132
Non-interactive	hsa:6096	D00246
Non-interactive	hsa:5241	D00105
Non-interactive	hsa:5914	D01294
Non-interactive	hsa:6257	D00182
Non-interactive	hsa:5465	D00954
Non-interactive	hsa:6097	D00088
Non-interactive	hsa:5241	D00163
Non-interactive	hsa:6097	D01689
Non-interactive	hsa:9971	D02367
Non-interactive	hsa:5916	D00554
Non-interactive	hsa:2103	D01217
Non-interactive	hsa:2908	D00067
Non-interactive	hsa:6256	D00596
Non-interactive	hsa:5916	D02217
Non-interactive	hsa:2099	D00690
Non-interactive	hsa:5916	D00627
Non-interactive	hsa:5468	D00094
Non-interactive	hsa:2103	D00088
Non-interactive	hsa:2101	D00462
Non-interactive	hsa:6095	D00962
Non-interactive	hsa:5914	D00961
Non-interactive	hsa:6256	D01115
Non-interactive	hsa:9970	D00730
Non-interactive	hsa:3174	D00312
Non-interactive	hsa:5465	D00462

Supplementary Information S2. The fingerprints for the 53 different drug codes occurring in Online Supporting Information S1. Each of these fingerprints is a 256-D vectors generated by the OpenBabel software downloaded from http://openbabel.org/ (Open Babel). See the text of the main paper for further explanation.

```
>D05341
00010000 01000000 00000000 00000000 00080000 00000000 00000000 00000000
00000000 00000000 00000000 40001000 04008008 00800000 00000000 00080008
00000000 00000000 00402000 00000001 00000000 00000a00 01000000 00000010
00000000 00000000 00000000 00000000 00000000 00000000 00020000 00000000

>D04066
00048000 63000008 6000010a 00010600 00081000 40000202 02040000 00000001
04000a00 00080840 08300000 40008000 00008a02 20a00000 01010021 001a0008
a0020200 02000100 02482000 00020201 00000100 08000d10 11004000 00402018
00000000 88010023 00120000 00000000 00000202 01060003 80020000 10020600

>D02367
00000000 81000020 00000000 00000000 00492000 00000000 00000000 00000402
00004000 00100000 00000080 40400000 00020000 00800004 00000400 00090008
00000000 00000020 02402000 00000001 00000008 00000000 01008000 00000010
00200000 00000000 00604000 80000000 00000000 01000200 24020800 00000000

>D02217
1c012000 11000809 2010090b 80010602 00181080 00808000 02040480 29100008
00400000 008808c1 08200200 d0018280 0080803a 02200081 21083181 00080008
10020000 82000008 04000000 00024201 00000000 58860a88 32004090 00500014
02804002 a4000010 00130000 50000645 020042a8 00060002 80000000 00c30e00

>D01689
00010002 81000020 00000508 00c90300 40280000 04400000 00007880 0a000402
01202000 00100020 00100000 4000d000 04028028 02800000 00000410 00280008
00820040 80000020 1241a000 00122001 00100001 00000b80 05008040 08000810
00200000 20000000 00404000 a4000000 00000200 01000002 24220000  02020000

>D01441
0001e202 00010800 80004100 44550610 09041004 04004000 00002800 02081200
00000100 00010840 00282004 6000d810 82008802 0010a002 80020440 800c0004
00060000 12006004 0c600000 10a00006 40100000 2c02a600 31812860 0240011c
000c0003 c0180000 08000600 800110e0 08c0810c 00460801 c0040800 200102a2

>D01387
00010042 81400020 00100100 00491300 403c1440 04200004 00000880 0a000402
01200000 01100020 00100000 4000d000 14028028 02800000 00000410 08289008
00820000 80000020 1341a000 00022001 00000021 10000a80 05008000 08520814
00200400 00000002 00404020 b4000020 00000200 0300000a 24a20000 02000200

>D01294
00000000 01000020 00400100 00010300 00582000 00000800 02000000 08000402
00004000 00100008 40000080 40008000 00028000 00800004 00000400 00091008
00820000 00000020 22402010 00000001 00000028 00000a00 01008000 00000010
00201400 00000500 00404000 80000008 00000200 01000202 24020800 00800010

>D01217
00010000 01040020 00000000 00000000 40000000 00000001 00000080 00000402
01004000 00108000 00000000 40001000 04028008 20000004 00000400 00000004
00000000 00000020 02092080 00000001 00000000 00000280 00008000 00000012
00200000 00000000 00404200 80000000 00000000 01000000 24020000 00000000
```

```
>D01161
00000000 67000008 20100102 00110200 00080000 40000082 02000000 00000001
05200800 00084840 08100000 40128000 00010002 00a40000 80000020 00080008
04000200 02000000 02402000 00020201 40000100 08000001 01004300 00f40010
00000000 80020001 00120020 90000000 0000000a 03020022 00020000 01010620

>D01132
10000202 00821a00 00024902 511d0290 0044b033 00004020 00400000 00000000
14800900 000a4851 08510004 60028000 08218800 00050000 02020480 0a0c400c
04020000 02000004 00214010 00000011 e0000101 08849a40 21800121 0240001d
00262404 80042000 48088040 04000001 04400204 00030072 d0000000 01018420

>D01115
00050040 81000020 00000900 80010300 00280080 00000000 00000280 00000402
01004000 00108008 00040200 40009000 04028028 02800004 00080404 00081008
00820000 00000120 12412000 0200a001 00000020 00001a80 0100a000 00000010
00200400 00000000 00404000 80000000 00000200 81000002 24020000 00000000

>D00965
00014006 01003810 40100120 00010e00 24000000 80000000 00000c04 00002009
14000000 00000a70 04280004 4000b004 04019080 09102000 20120140 00000010
00040000 02402000 08000000 00000011 48120100 38050600 20022000 0040001a
00140000 c0000000 00000000 900000a0 0a408000 00060001 20104000 04004200

>D00962
04052000 01000008 20000108 00010602 00281080 00008000 82040080 09000400
00400000 000a0841 08200100 5000d000 14808022 02000000 01001003 00080008
10000400 02000000 01400010 00020001 00000000 18040b80 17000001 00400010
00000000 a8000000 00522000 40000001 0040028a 00060002 04000000 00020600

>D00961
00004002 04808a00 40121100 02910640 241000a0 00000010 00004c00 00000000
00000000 00804850 20683004 6100d024 00118000 01349000 82220040 00080008
04440000 02000000 08804000 00402010 c4100010 18000602 22802680 08540019
08042200 80000000 08000000 90000081 00408000 00068421 10100000 05010220

>D00956
00010040 01000028 00000100 00010300 00080000 00000000 00000080 00000402
01004800 00188840 08100000 40009000 04028008 00800004 00000400 00081008
00820000 02000020 02412008 00000001 00000120 08000a80 01008040 00400010
00200c00 80000000 00404000 80000000 00000200 01020002 24020000 00000400

>D00954
00010000 01000020 00000000 00000000 00482000 00000000 00000080 00000402
01004000 00108000 00000080 40001000 04028008 00800004 00000400 00090008
00000000 00000020 02412000 00000001 00000008 00000280 01008000 00000010
00200000 00000000 00404000 80000000 00000000 01000200 24020800 00000000

>D00951
00010000 01000020 00000100 00010300 00080000 00000000 00000080 00000402
01004000 00108000 00000000 4000d000 04028008 00800004 00000400 00081008
00820000 80000020 02412000 00000001 00000020 00000a80 01008000 00000014
00200400 00000000 00404000 80000000 00000200 01000002 24020000 02000000

>D00950
00010000 01000020 00000000 00000000 00482000 00000000 00000080 00000402
01004000 00108000 00000080 40001000 04028008 00800004 00000400 00090008
00000000 00000020 02412000 00000001 00000008 00000280 01008000 00000010
00200000 00000000 00404000 80000000 00000000 01000200 24020800 00000000
```

```
>D00930
00000000 01040000 00000800 00000000 40080000 00000001 00000000 00000402
00000000 00100008 00000200 40000000 00020000 02800000 00000404 0008000c
00000000 00000000 03402080 00000001 00000000 00000000 01008000 00000012
00200000 00000000 00404000 80000000 00000000 01000000 24020000 04000000

>D00898
00000000 00040008 20000100 00010200 00000000 00800001 82000081 00000400
00000800 00980840 08100000 40008000 00000002 00000000 00000002 00000008
00000400 02100000 00000080 00020801 00000100 08100000 00008001 00400010
00000000 80000000 00522000 40000000 00400000 00020002 04000000 00000500

>D00730
00000002 00000800 00000100 80090300 00000010 00004000 00000000 00000000
00000100 00000840 00000004 40008000 00008000 00010000 00000040 02081008
00020000 02000000 00400000 00000001 40000020 08020a20 21000000 00400014
00000400 80002000 00080000 00000000 00000200 00060002 c0000000 02000000

>D00690
001b0002 81200820 00080101 00490300 40380010 04004000 00000080 02200402
03004100 02100000 00102520 4000f000 46028428 00810000 80000430 02081008
00824000 82002020 12412000 00022101 00000021 88040b80 0100a000 04480a14
20200440 28002000 04484010 88000008 00800285 01020002 e4024000 02822800

>D00627
0800e00a 00800a01 00101500 10050610 000c0061 0000c000 00000000 00081000
80500100 100018d0 0110800c 40818800 82009880 00608000 200300c0 020c000c
00020000 06802004 04680008 50410005 c0100008 38028a30 31880041 81500414
00040000 c0000000 00000040 00000040 0440400c 00c60c08 c00c0804 200004a0

>D00596
04006002 00001328 20107308 00110606 00051080 00000800 02040000 00000400
01400800 230948c1 08340005 40009300 0481b802 000c0000 01001001 000c000c
04040410 02004200 08210018 00a20101 40120108 1c048e04 32800900 02600018
00001800 80001020 08520080 140000c0 00008208 0006081b 04005010 83010600

>D00586
00004002 00001810 40100100 00010600 24000000 80000000 00000c00 00000000
14000000 00000870 04280004 40009004 00018080 01100000 20120140 00000010
00040000 02000000 08000000 00000010 48120000 18040600 20002000 00400010
00040000 c0000000 00000000 900000a0 02408000 00060001 00100000 04000200

>D00585
00010202 01040820 00000102 40010200 40482000 00800001 00200081 00000402
01004800 00988848 08300084 40009008 04028008 20a08004 00000442 0009000c
00040000 02100020 0a692080 02000801 40000108 08000280 21008800 00400012
00200000 80000000 00404200 80000080 00008002 81060202 24020804 00000400

>D00577
00000000 00000008 20000300 00010200 00000000 00000000 82000080 00000402
00000800 00180840 08100100 40008000 00000002 00000000 00000002 00000008
00000400 02000000 00000000 00020001 00000100 18000000 00008001 00400010
01000000 80000000 00522000 40000000 00400000 00020002 04000000 00000400

>D00565
04008000 00000808 20000109 00010200 00001000 00000000 02040000 01000040
00002000 00080840 08040000 4000d800 02808026 00000000 01200001 00080008
00020000 82000000 00000002 00020000 00000020 08020b00 05000000 08400014
00002400 a0000000 00160008 40800020 00000280 00020002 80000000 00020400
```

```
>D00554
00000000 63000008 20000102 00010200 00482000 40000002 02000000 00000001
00000800 00080840 08100080 40008000 00000002 00a00000 00000020 00090008
00000200 02000000 02402000 00020201 00000108 08000000 01004000 00400010
00000000 80000001 00120000 00000000 00000002 01020202 00020800 00000400

>D00506
00000000 01001000 00100100 00010e00 00000000 00000000 00000004 00002004
00000900 000809c0 08300000 40009000 0d009000 00000000 40000000 00000000
00000008 02000001 00000010 00000001 00020100 182d0600 00000000 81400018
00012000 80000000 00000000 10000000 04000000 00060213 00082000 00000600

>D00462
00010040 01000000 00000100 00010300 80080000 00000000 00000000 00000000
00000000 00000000 00000000 40009000 04008028 00800000 00000000 00081008
00820000 00000000 02402000 00000001 00000020 00000a00 01000000 00000010
00000400 00000000 00000000 00000000 00000200 01000002 00020000 00000000

>D00443
00050040 01000120 00010100 00110304 00080002 00000000 00000081 00000402
05a04000 0010c001 00000000 40029100 04038008 00840004 00080400 00081008
04820000 000000a0 02412000 00100801 40000020 00000a80 01008300 00200010
00200400 00000000 04404000 80000000 00000200 01001822 24020000 81010000

>D00348
00000400 01040020 00000000 00000000 40000000 00000001 00000000 00000402
01004000 00100000 00000000 40000000 00028000 20020004 00000400 0008000c
00000000 00000020 02092090 00000001 00000000 00000a00 00408000 00000012
00201000 00000000 00404000 80000000 00000000 00000000 24820000 00000000

>D00327
00010000 81400020 00100000 00001000 00281400 00000000 00000080 00000402
01004000 00108000 00000000 40001000 04028008 00800004 00000400 00080008
00000000 00000020 02412000 00002001 00000000 00000280 01008000 00520010
00200000 00000002 00404020 90000000 00000000 03000000 24020000 00000200

>D00316
00080400 40040008 20000308 00010200 42001001 00000001 82040080 00000400
01000100 00100840 40000000 40008000 00008002 20220000 01000003 0008200c
00020400 02400000 20090090 00020200 00100010 08000a00 00408001 00400010
01001400 80000000 00422000 40000000 00404340 08220000 04800000 01000010

>D00312
00010000 63020008 20000302 00410200 20000020 40002002 82000000 00002801
00000800 00880840 08102000 40809000 0400800a 00200002 00000020 00080008
02000a00 02000000 02802000 00020201 00000100 08000200 00004010 00400011
00100000 80000001 00120000 00000000 00000002 01020402 00020000 00010c20

>D00299
00000000 01040000 00000000 00000000 40080000 00000001 00000000 00000402
00000000 00100008 00000200 40000000 00020000 00800000 00000404 0008000c
00000000 00000000 02402080 00000001 00000000 00000000 01008000 00000012
00200000 00000000 00404000 80000000 00000000 01000000 24020000 04000000

>D00279
04008000 00000008 20000008 00010200 00001000 00000000 02000000 00000040
00002000 00000840 00040000 4000d800 02808006 00000000 01200001 00080008
00020000 80000400 00000002 00020000 00000020 08000b00 05000000 08000010
00002400 00000000 00100008 00800020 00000200 00020000 00000000 00020000
```

```
>D00246
00010042 81000020 00000000 00490100 403c0040 04200004 00000880 0a000402
01200000 01100020 00100000 4000d000 14028028 02800000 00000410 08289008
00800000 80000020 1341a000 00022001 00000021 00000280 05008000 08000814
00200400 00000000 00404000 a4000020 00000200 01000000 24a20000 00000000

>D00211
0400520f c3048808 20910109 d4010781 70485041 42020041 42430000 03045642
01210b00 11580849 483c021c 6000d9f8 8082c022 22912000 030e0445 3838701e
28860084 b6008101 0d496c90 00022e01 401c4120 38030e88 6104a020 02408016
00004420 80400a00 605f4858 a0401084 0945c358 000e020b e402a400 06010618

>D00188
00000400 81040000 00000000 00000000 40080000 00000001 00000000 00000400
00000000 00100008 00000200 40000000 00020000 00800000 00000404 0008000c
00000000 00000000 02402080 00000001 00000000 00000000 01008000 00000012
00000000 00000000 00404000 80000000 00000000 01000000 24820000 04000000

>D00187
00000400 81040000 00000000 00000000 40080000 00000001 00000000 00000402
00000000 00100008 00000200 40000000 00020000 00800000 00000404 0008000c
00000000 00000000 02402080 00000001 00000000 00000000 01008000 00000012
00200000 00000000 00404000 80000000 00000000 01000000 24820000 04000000

>D00182
00010000 01000020 00000000 00000000 00482000 00000000 00000080 00000402
01004000 00108000 00000080 40001000 04028008 00800004 00000400 00090008
00000000 00000020 02412000 00000001 00000008 00000280 01008000 00000010
00200000 00000000 00404000 80000000 00000000 01000200 24020800 00000000

>D00163
00010000 01000000 00000000 00000000 00080000 00000000 00000000 00000000
00000000 00000000 00000000 40001000 04008008 02800000 00000000 00080008
00000000 00000000 02402000 00000001 00000000 00000a00 01000000 00000010
00000000 00000000 00000000 00000000 00000000 01000000 00020000 00000000

>D00143
00010000 01000020 00000800 00000000 00080000 00000000 00000000 00000402
00004000 00100008 00000200 40001000 04028008 00800004 00000404 00080008
00000000 00000020 02402000 00000001 00000000 00000200 01008000 00000010
00200000 00000000 00404000 80000000 00000000 01000000 24020000 00000000

>D00105
00000000 63000008 20000102 00010200 00080000 40000002 02000000 00000001
00000800 00080840 08100000 40008000 00000002 00a00000 00000020 00080008
00000200 02000000 02402000 00020201 00000100 08000000 01004000 00400010
00000000 80000001 00120000 00000000 00000002 01020002 00020000 00000400

>D00094
00000400 01040020 00000000 00000000 40000000 00000001 00000000 00000402
01004000 00100000 00000000 40000000 00028000 20020004 00000400 0008000c
00000000 00000020 02092090 00000001 00000000 00000a00 00408000 00000012
00201000 00000000 00404000 80000000 00000000 00000000 24820000 00000000

>D00088
00010000 81000020 00000000 00000000 00280000 00000000 00000080 00000402
01004000 00108000 00000000 40005000 04028028 00800004 00000400 00080008
00000000 00000020 03412000 00002001 00000000 00000280 01008000 00000010
00200000 00000000 00404000 80000000 00000000 01000000 24020000 00000000
```

>D00075
00010000 01000020 00000000 00000000 00080000 00000000 00000080 00000402
01004000 00108000 00000000 40001000 04028008 00800004 00000400 00080008
00000000 00000020 02412000 00000001 00000000 00000280 01008000 00000010
00200000 00000000 00404000 80000000 00000000 01000000 24020000 00000000

>D00067
00010000 63000008 20000102 00010200 00000000 40000002 02000000 00000001
00000800 00080840 08100000 40009000 0400800a 00200000 00000020 00080008
00000200 02000000 02002000 00020201 00000100 08000200 00004000 00400010
00000000 80000001 00120000 00000000 00000002 01020002 00020000 00000400

>D00066
00010000 01000020 00000000 00000000 00000000 00000000 00000080 00000402
01004000 00108000 00000000 40001000 04028008 00000004 00000400 00000000
00000000 00000020 02012000 00000001 00000000 00000280 00008000 00000010
00200000 00000000 00404000 80000000 00000000 01000000 24020000 00000000

>D00040
00000000 01000020 00000800 00000000 00080000 00000000 00000000 00000402
00004000 00100008 00000200 40000000 00020000 00800004 00000404 00080008
00000000 00000020 02402000 00000001 00000000 00000000 01008000 00000010
00200000 00000000 00404000 80000000 00000000 01000000 24020000 00000000

Supplementary Information S3. The protein sequences for the 25 different nuclear receptors occurring in Supplementary Information S1.

>hsa:2099
MTMTLHTKASGMALLHQIQGNELEPLNRPQLKIPLERPLGEVYLDSSKPAVYNYPEGAAY
EFNAAAAANAQVYGQTGLPYGPGSEAAAFGSNGLGGFPPLNSVSPSPLMLLHPPPQLSPF
LQPHGQQVPYYLENEPSGYTVREAGPPAFYRPNSDNRRQGGRERLASTNDKGSMAMESAK
ETRYCAVCNDYASGYHYGVWSCEGCKAFFKRSIQGHNDYMCPATNQCTIDKNRRKSCQAC
RLRKCYEVGMMKGGIRKDRRGGRMLKHKRQRDDGEGRGEVGSAGDMRAANLWPSPLMIKR
SKKNSLALSLTADQMVSALLDAEPPILYSEYDPTRPFSEASMMGLLTNLADRELVHMINW
AKRVPGFVDLTLHDQVHLLECAWLEILMIGLVWRSMEHPGKLLFAPNLLLDRNQGKCVEG
MVEIFDMLLATSSRFRMMNLQGEEFVCLKSIILLNSGVYTFLSSTLKSLEEKDHIHRVLD
KITDTLIHLMAKAGLTLQQQHQRLAQLLLILSHIRHMSNKGMEHLYSMKCKNVVPLYDLL
LEMLDAHRLHAPTSRGGASVEETDQSHLATAGSTSSHSLQKYYITGEAEGFPATV

>hsa:190
MAGENHQWQGSILYNMLMSAKQTRAAPEAPETRLVDQCWGCSCGDEPGVGREGLLGGRNV
ALLYRCCFCGKDHPRQGSILYSMLTSAKQTYAAPKAPEATLGPCWGCSCGSDPGVGRAGL
PGGRPVALLYRCCFCGEDHPRQGSILYSLLTSSKQTHVAPAAPEARPGGAWWDRSYFAQR
PGGKEALPGGRATALLYRCCFCGEDHPQQGSTLYCVPTSTNQAQAAPEERPRAPWWDTSS
GALRPVALKSPQVVCEAASAGLLKTLRFVKYLPCFQVLPLDQQLVLVRNCWASLLMLELA
QDRLQFETVEVSEPSMLQKILTTRRRETGGNEPLPVPTLQHHLAPPAEARKVPSASQVQA
IKCFLSKCWSLNISTKEYAYLKGTVLFNPDVPGLQCVKYIQGLQWGTQQILSEHTRMTHQ
GPHDRFIELNSTLFLLRFINANVIAELFFRPIIGTVSMDDMMLEMLCTKI

>hsa:2100
MDIKNSPSSLNSPSSYNCSQSILPLEHGSIYIPSSYVDSHHEYPAMTFYSPAVMNYSIPS
NVTNLEGGPGRQTTSPNVLWPTPGHLSPLVVHRQLSHLYAEPQKSPWCEARSLEHTLPVN
RETLKRKVSGNRCASPVTGPGSKRDAHFCAVCSDYASGYHYGVWSCEGCKAFFKRSIQGH
NDYICPATNQCTIDKNRRKSCQACRLRKCYEVGMVKCGSRRERCGYRLVRRQRSADEQLH
CAGKAKRSGGHAPRVRELLLDALSPEQLVLTLLEAEPPHVLISRPSAPFTEASMMMSLTK
LADKELVHMISWAKKIPGFVELSLFDQVRLLESCWMEVLMMGLMWRSIDHPGKLIFAPDL
VLDRDEGKCVEGILEIFDMLLATTSRFRELKLQHKEYLCVKAMILLNSSMYPLVTATQDA
DSSRKLAHLLNAVTDALVWVIAKSGISSQQQSMRLANLLMLLSHVRHARAEKASQTLTSF
GMKMETLLPEATMEQ

>hsa:2101
MSSQVVGIEPLYIKAEPASPDSPKGSSETETEPPVALAPGPAPTRCLPGHKEEEDGEGAG
PGEQGGGKLVLSSLPKRLCLVCGDVASGYHYGVASCEACKAFFKRTIQGSIEYSCPASNE
CEITKRRRKACQACRFTKCLRVGMLKEGVRLDRVRGGRQKYKRRPEVDPLPFPGPFPAGP
LAVAGGPRKTAAPVNALVSHLLVVEPEKLYAMPDPAGPDGHLPAVATLCDLFDREIVVTI
SWAKSIPGFSSLSLSDQMSVLQSVWMEVLVLGVAQRSLPLQDELAFAEDLVLDEEGARAA
GLGELGAALLQLVRRLQALRLEREEYVLLKALALANSDSVHIEDAEAVEQLREALHEALL
EYEAGRAGPGGGAERRRAGRLLLTLPLLRQTAGKVLAHFYGVKLEGKVPMHKLFLEMLEA
MMD

>hsa:2103
MSSDDRHLGSSCGSFIKTEPSSPSSGIDALSHHSPSGSSDASGGFGLALGTHANGLDSPP
MFAGAGLGGTPCRKSYEDCASGIMEDSAIKCEYMLNAIPKRLCLVCGDIASGYHYGVASC
EACKAFFKRTIQGNIEYSCPATNECEITKRRRKSCQACRFMKCLKVGMLKEGVRLDRVRG
GRQKYKRRLDSESSPYLSLQISPPAKKPLTKIVSYLLVAEPDKLYAMPPPGMPEGDIKAL
TTLCDLADRELVVIIGWAKHIPGFSSLSLGDQMSLLQSAWMEILILGIVYRSLPYDDKLV
YAEDYIMDEEHSRLAGLLELYRAILQLVRRYKKLKVEKEEFVTLKALALANSDSMYIEDL
EAVQKLQDLLHEALQDYELSQRHEEPWRTGKLLLTLPLLRQTAAKAVQHFYSVKLQGKVP
MHKLFLEMLEAKVGQEQLRGSPKDERMSSHDGKCPFQSAAFTSRDQSNSPGIPNPRPSSP
TPLNERGRQISPSTRTPGGQGKHLWLTM

>hsa:2104
MSNKDRHIDSSCSSFIKTEPSSPASLTDSVNHHSPGGSSDASGSYSSTMNGHQNGLDSPP
LYPSAPILGGSGPVRKLYDDCSSTIVEDPQTKCEYMLNSMPKRLCLVCGDIASGYHYGVA
SCEACKAFFKRTIQGNIEYSCPATNECEITKRRRKSCQACRFMKCLKVGMLKEGVRLDRV
RGGRQKYKRRIDAENSPYLNPQLVQPAKKPYNKIVSHLLVAEPEKIYAMPDPTVPDSDIK
ALTTLCDLADRELVVIIGWAKHIPGFSTLSLADQMSLLQSAWMEILILGVVYRSLSFEDE
LVYADDYIMDEDQSKLAGLLDLNNAILQLVKKYKSMKLEKEEFVTLKAIALANSDSMHIE
DVEAVQKLQDVLHEALQDYEAGQHMEDPRRAGKMLMTLPLLRQTSTKAVQHFYNIKLEGK
VPMHKLFLEMLEAKV

>hsa:2908
MDSKESLTPGREENPSSVLAQERGDVMDFYKTLRGGATVKVSASSPSLAVASQSDSKQRR
LLVDFPKGSVSNAQQPDLSKAVSLSMGLYMGETETKVMGNDLGFPQQGQISLSSGETDLK
LLEESIANLNRSTSVPENPKSSASTAVSAAPTEKEFPKTHSDVSSEQQHLKGQTGTNGGN
VKLYTTDQSTFDILQDLEFSSGSPGKETNESPWRSDLLIDENCLLSPLAGEDDSFLLEGN
SNEDCKPLILPDTKPKIKDNGDLVLSSPSNVTLPQVKTEKEDFIELCTPGVIKQEKLGTV
YCQASFPGANIIGNKMSAISVHGVSTSGGQMYHYDMNTASLSQQQDQKPIFNVIPPIPVG
SENWNRCQGSGDDNLTSLGTLNFPGRTVFSNGYSSPSMRPDVSSPPSSSSTATTGPPPKL
CLVCSDEASGCHYGVLTCGSCKVFFKRAVEGQHNYLCAGRNDCIIDKIRRKNCPACRYRK
CLQAGMNLEARKTKKKIKGIQQATTGVSQETSENPGNKTIVPATLPQLTPTLVSLLEVIE
PEVLYAGYDSSVPDSTWRIMTTLNMLGGRQVIAAVKWAKAIPGFRNLHLDDQMTLLQYSW
MFLMAFALGWRSYRQSSANLLCFAPDLIINEQRMTLPCMYDQCKHMLYVSSELHRLQVSY
EEYLCMKTLLLLSSVPKDGLKSQELFDEIRMTYIKELGKAIVKREGNSSQNWQRFYQLTK
LLDSMHEVVENLLNYCFQTFLDKTMSIEFPEMLAEIITNQIPKYSNGNIKKLLFHQK

>hsa:3174
MDMANYSEVLDPTYTTLEFETMQILYNSSDSSAPETSMNTTDNGVNCLCAICGDRATGKH
YGASSCDGCKGFFRRSIRKSHVYSCRFSRQCVVDKDKRNQCRYCRLRKCFRAGMKKEAVQ
NERDRISTRRSTFDGSNIPSINTLAQAEVRSRQISVSSPGSSTDINVKKIASIGDVCESM
KQQLLVLVEWAKYIPAFCELPLDDQVALLRAHAGEHLLLGATKRSMMYKDILLLGNNYVI
HRNSCEVEISRVANRVLDELVRPFQEIQIDDNEYACLKAIVFFDPDAKGLSDPVKIKNMR
FQVQIGLEDYINDRQYDSRGRFGELLLLLPTLQSITWQMIEQIQFVKLFGMVKIDNLLQE
MLLGGASNDGSHLHHPMHPHLSQDPLTGQTILLGPMSTLVHADQISTPETPLPSPPQGSG
QEQYKIAANQASVISHQHLSKQKQL

>hsa:367
MEVQLGLGRVYPRPPSKTYRGAFQNLFQSVREVIQNPGPRHPEAASAAPPGASLLLLQQQ
QQQQQQQQQQQQQQQQQQQQQETSPRQQQQQQGEDGSPQAHRRGPTGYLVLDEEQQPSQPQ
SALECHPERGCVPEPGAAVAASKGLPQQLPAPPDEDDSAAPSTLSLLGPTFPGLSSCSAD
LKDILSEASTMQLLQQQQQEAVSEGSSSGRAREASGAPTSSKDNYLGGTSTISDNAKELC
KAVSVSMGLGVEALEHLSPGEQLRGDCMYAPLLGVPPAVRPTPCAPLAECKGSLLDDSAG
KSTEDTAEYSPFKGGYTKGLEGESLGCSGSAAAGSSGTLELPSTLSLYKSGALDEAAAYQ
SRDYYNFPLALAGPPPPPPPPHPHARIKLENPLDYGSAWAAAAAQCRYGDLASLHGAGAA
GPGSGSPSAAASSSWHTLFTAEEGQLYGPCGGGGGGGGGGGGGGGGGGGGGGGGEAGAVAP
YGYTRPPQGLAGQESDFTAPDVWYPGGMVSRVPYPSPTCVKSEMGPWMDSYSGPYGDMRL
ETARDHVLPIDYYFPPQKTCLICGDEASGCHYGALTCGSCKVFFKRAAEGKQKYLCASRN
DCTIDKFRRKNCPSCRLRKCYEAGMTLGARKLKKLGNLKLQEEGEASSTTSPTEETTQKL

TVSHIEGYECQPIFLNVLEAIEPGVVCAGHDNNQPDSFAALLSSLNELGERQLVHVVKWA
KALPGFRNLHVDDQMAVIQYSWMGLMVFAMGWRSFTNVNSRMLYFAPDLVFNEYRMHKSR
MYSQCVRMRHLSQEFGWLQITPQEFLCMKALLLFSIIPVDGLKNQKFFDELRMNYIKELD
RIIACKRKNPTSCSRRFYQLTKLLDSVQPIARELHQFTFDLLIKSHMVSVDFPEMMAEII
SVQVPKILSGKVKPIYFHTQ

>hsa:4306
METKGYHSLPEGLDMERRWGQVSQAVERSSLGPTERTDENNYMEIVNVSCVSGAIPNNST
QGSSKEKQELLPCLQQDNNRPGILTSDIKTELESKELSATVAESMGLYMDSVRDADYSYE
QQNQQGSMSPAKIYQNVEQLVKFYKGNGHRPSTLSCVNTPLRSFMSDSGSSVNGGVMRAV
VKSPIMCHEKSPSVCSPLNMTSSVCSPAGINSVSSTTASFGSFPVHSPITQGTPLTCSPN
VENRGSRSHSPAHASNVGSPLSSPLSSMKSSISSPPSHCSVKSPVSSPNNVTLRSSVSSP
ANINNSRCSVSSPSNTNNRSTLSSPAASTVGSICSPVNNAFSYTASGTSAGSSTLRDVVP
SPDTQEKGAQEVPFPKTEEVESAISNGVTGQLNIVQYIKPEPDGAFSSSCLGGNSKINSD
SSFSVPIKQESTKHSCSGTSFKGNPTVNPFPFMDGSYFSFMDDKDYYSLSGILGPPVPGF
DGNCEGSGFPVGIKQEPDDGSYYPEASIPSSAIVGVNSGGQSFHYRIGAQGTISLSRSAR
DQSFQHLSSFPPVNTLVESWKSHGDLSSRRSDGYPVLEYIPENVSSSTLRSVSTGSSRPS
KICLVCGDEASGCHYGVVTCGSCKVFFKRAVEGQHNYLCAGRNDCIIDKIRRKNCPACRL
QKCLQAGMNLGARKSKKLGKLKGIHEEQPQQQQPPPPPPPPQSPEEGTTYIAPAKEPSVN
TALVPQLSTISRALTPSPVMVLENIEPEIVYAGYDSSKPDTAENLLSTLNRLAGKQMIQV
VKWAKVLPGFKNLPLEDQITLIQYSWMCLSSFALSWRSYKHTNSQFLYFAPDLVFNEEKM
HQSAMYELCQGMHQISLQFVRLQLTFEEYTIMKVLLLLSTIPKDGLKSQAAFEEMRTNYI
KELRKMVTKCPNNSGQSWQRFYQLTKLLDSMHDLVSDLLEFCFYTFRESHALKVEFPAML
VEIISDQLPKVESGNAKPLYFHRK

>hsa:5241
MTELKAKGPRAPHVAGGPPSPEVGSPLLCRPAAGPFPGSQTSDTLPEVSAIPISLDGLLF
PRPCQGQDPSDEKTQDQQSLSDVEGAYSRAEATRGAGGSSSSPPEKDSGLLDSVLDTLLA
PSGPGQSQPSPPACEVTSSWCLFGPELPEDPPAAPATQRVLSPLMSRSGCKVGDSSGTAA
AHKVLPRGLSPARQLLLPASESPHWSGAPVKPSPQAAAVEVEEEDGSESEESAGPLLKGK
PRALGGAAAGGGAAAVPPGAAAGGVALVPKEDSRFSAPRVALVEQDAPMAPGRSPLATTV
MDFIHVPILPLNHALLAARTRQLLEDESYDGGAGAASAFAPPRSSPCASSTPVAVGDFPD
CAYPPDAEPKDDAYPLYSDFQPPALKIKEEEEGAEASARSPRSYLVAGANPAAFPDFPLG
PPPPLPPRATPSRPGEAAVTAAPASASVSSASSSGSTLECILYKAEGAPPQQGPFAPPPC
KAPGASGCLLPRDGLPSTSASAAAAGAAPALYPALGLNGLPQLGYQAAVLKEGLPQVYPP
YLNYLRPDSEASQSPQYSFESLPQKICLICGDEASGCHYGVLTCGSCKVFFKRAMEGQHN
YLCAGRNDCIVDKIRRKNCPACRLRKCCQAGMVLGGRKFKKFNKVRVVRALDAVALPQPV
GVPNESQALSQRFTFSPGQDIQLIPPLINLLMSIEPDVIYAGHDNTKPDTSSSLLTSLNQ
LGERQLLSVVKWSKSLPGFRNLHIDDQITLIQYSWMSLMVFGLGWRSYKHVSGQMLYFAP
DLILNEQRMKESSFYSLCLTMWQIPQEFVKLQVSQEEFLCMKVLLLLNTIPLEGLRSQTQ
FEEMRSSYIRELIKAIGLRQKGVVSSSQRFYQLTKLLDNLHDLVKQLHLYCLNTFIQSRA
LSVEFPEMMSEVIAAQLPKILAGMVKPLLFHKK

>hsa:5465
MVDTESPLCPLSPLEAGDLESPLSEEFLQEMGNIQEISQSIGEDSSGSFGFTEYQYLGSC
PGSDGSVITDTLSPASSPSSVTYPVVPGSVDESPSGALNIECRICGDKASGYHYGVHACE
GCKGFFRRTIRLKLVYDKCDRSCKIQKKNRNKCQYCRFHKCLSVGMSHNAIRFGRMPRSE
KAKLKAEILTCEHDIEDSETADLKSLAKRIYEAYLKNFNMNKVKARVILSGKASNNPPFV
IHDMETLCMAEKTLVAKLVANGIQNKEAEVRIFHCCQCTSVETVTELTEFAKAIPGFANL
DLNDQVTLLKYGVYEAIFAMLSSVMNKDGMLVAYGNGFITREFLKSLRKPFCDIMEPKFD
FAMKFNALELDDSDISLFVAAIICCGDRPGLLNVGHIEKMQEGIVHVLRLHLQSNHPDDI
FLFPKLLQKMADLRQLVTEHAQLVQIIKKTESDAALHPLLQEIYRDMY

>hsa:5468
MTMVDTEMPFWPTNFGISSVDLSVMEDHSHSFDIKPFTTVDFSSISTPHYEDIPFTRTDP
VVADYKYDLKLQEYQSAIKVEPASPPYYSEKTQLYNKPHEEPSNSLMAIECRVCGDKASG
FHYGVHACEGCKGFFRRTIRLKLIYDRCDLNCRIHKKSRNKCQYCRFQKCLAVGMSHNAI
RFGRMPQAEKEKLLAEISSDIDQLNPESADLRALAKHLYDSYIKSFPLTKAKARAILTGK
TTDKSPFVIYDMNSLMMGEDKIKFKHITPLQEQSKEVAIRIFQGCQFRSVEAVQEITEYA
KSIPGFVNLDLNDQVTLLKYGVHEIIYTMLASLMNKDGVLISEGQGFMTREFLKSLRKPF
GDFMEPKFEFAVKFNALELDDSDLAIFIAVIILSGDRPGLLNVKPIEDIQDNLLQALELQ
LKLNHPESSQLFAKLLQKMTDLRQIVTEHVQLLQVIKKTETDMSLHPLLQEIYKDLY

>hsa:5914
MASNSSSCPTPGGGHLNGYPVPPYAFFFPPMLGGLSPPGALTTLQHQLPVSGYSTPSPAT
IETQSSSSEEIVPSPPSPPPLPRIYKPCFVCQDKSSGYHYGVSACEGCKGFFRRSIQKNM
VYTCHRDKNCIINKVTRNRCQYCRLQKCFEVGMSKESVRNDRNKKKKEVPKPECSESYTL
TPEVGELIEKVRKAHQETFPALCQLGKYTTNNSSEQRVSLDIDLWDKFSELSTKCIIKTV

EFAKQLPGFTTLTIADQITLLKAACLDILILRICTRYTPEQDTMTFSDGLTLNRTQMHNA
GFGPLTDLVFAFANQLLPLEMDDAETGLLSAICLICGDRQDLEQPDRVDMLQEPLLEALK
VYVRKRRPSRPHMFPKMLMKITDLRSISAKGAERVITLKMEIPGSMPPLIQEMLENSEGL
DTLSGQPGGGGRDGGGLAPPPGSCSPSLSPSSNRSSPATHSP

>hsa:5915
MFDCMDVLSVSPGQILDFYTASPSSCMLQEKALKACFSGLTQTEWQHRHTAQSIETQSTS
SEELVPSPPSPLPPPRVYKPCFVCQDKSSGYHYGVSACEGCKGFFRRSIQKNMIYTCHRD
KNCVINKVTRNRCQYCRLQKCFEVGMSKESVRNDRNKKKKETSKQECTESYEMTAELDDL
TEKIRKAHQETFPSLCQLGKYTTNSSADHRVRLDLGLWDKFSELATKCIIKIVEFAKRLP
GFTGLTIADQITLLKAACLDILILRICTRYTPEQDTMTFSDGLTLNRTQMHNAGFGPLTD
LVFTFANQLLPLEMDDTETGLLSAICLICGDRQDLEEPTKVDKLQEPLLEALKIYIRKRR
PSKPHMFPKILMKITDLRSISAKGAERVITLKMEIPGSMPPLIQEMLENSEGHEPLTPSS
SGNTAEHSPSISPSSVENSGVSQSPLVQ

>hsa:5916
MATNKERLFAAGALGPGSGYPGAGFPFAFPGALRGSPPFEMLSPSFRGLGQPDLPKEMAS
LSVETQSTSSEEMVPSSPSPPPPPRVYKPCFVCNDKSSGYHYGVSSCEGCKGFFRRSIQK
NMVYTCHRDKNCIINKVTRNRCQYCRLQKCFEVGMSKEAVRNDRNKKKKEVKEEGSPDSY
ELSPQLEELITKVSKAHQETFPSLCQLGKYTTNSSADHRVQLDLGLWDKFSELATKCIIK
IVEFAKRLPGFTGLSIADQITLLKAACLDILMLRICTRYTPEQDTMTFSDGLTLNRTQMH
NAGFGPLTDLVFAFAGQLLPLEMDDTETGLLSAICLICGDRMDLEEPEKVDKLQEPLLEA
LRLYARRRRPSQPYMFPRMLMKITDLRGISTKGAERAITLKMEIPGPMPPLIREMLENPE
MFEDDSSQPGPHPNASSEDEVPGGQGKGGLKSPA

>hsa:6095
MNEGAPGDSDLETEARVPWSIMGHCLRTGQARMSATPTPAGEGARSSSTCSSLSRLFWSQ
LEHINWDGATAKNFINLREFFSFLLPALRKAQIEIIPCKICGDKSSGIHYGVITCEGCKG
FFRRSQQSNATYSCPRQKNCLIDRTSRNRCQHCRLQKCLAVGMSRDAVKFGRMSKKQRDS
LYAEVQKHRMQQQQRDHQQQPGEAEPLTPTYNISANGLTELHDDLSNYIDGHTPEGSKAD
SAVSSFYLDIQPSPDQSGLDINGIKPEPICDYTPASGFFPYCSFTNGETSPTVSMAELEH
LAQNISKSHLETCQYLREELQQITWQTFLQEEIENYQNKQREVMWQLCAIKITEAIQYVV
EFAKRIDGFMELCQNDQIVLLKAGSLEVVFIRMCRAFDSQNNTVYFDGKYASPDVFKSLG
CEDFISFVFEFGKSLCSMHLTEDEIALFSAFVLMSADRSWLQEKVKIEKLQQKIQLALQH
VLQKNHREDGILTKLICKVSTLRALCGRHTEKLMAFKAIYPDIVRLHFPPLYKELFTSEF
EPAMQIDG

>hsa:6096
MRAQIEVIPCKICGDKSSGIHYGVITCEGCKGFFRRSQQNNASYSCPRQRNCLIDRTNRN
RCQHCRLQKCLALGMSRDAVKFGRMSKKQRDSLYAEVQKHQQRLQEQRQQQSGEAEALAR
VYSSSISNGLSNLNNETSGTYANGHVIDLPKSEGYYNVDSGQPSPDQSGLDMTGIKQIKQ
EPIYDLTSVPNLFTYSSFNNGQLAPGITMTEIDRIAQNIIKSHLETCQYTMEELHQLAWQ
THTYEEIKAYQSKSREALWQQCAIQITHAIQYVVEFAKRITGFMELCQNDQILLLKSGCL
EVVLVRMCRAFNPLNNTVLFEGKYGGMQMFKALGSDDLVNEAFDFAKNLCSLQLTEEEIA
LFSSAVLISPDRAWLIEPRKVQKLQEKIYFALQHVIQKNHLDDETLAKLIAKIPTITAVC
NLHGEKLQVFKQSHPEIVNTLFPPLYKELFNPDCATGCK

>hsa:6097
MRTQIEVIPCKICGDKSSGIHYGVITCEGCKGFFRRSQRCNAAYSCTRQQNCPIDRTSRN
RCQHCRLQKCLALGMSRDAVKFGRMSKKQRDSLHAEVQKQLQQRQQQQQEPVVKTPPAGA
QGADTLTYTLGLPDGQLPLGSSPDLPEASACPPGLLKASGSGPSYSNNLAKAGLNGASCH
LEYSPERGKAEGRESFYSTGSQLTPDRCGLRFEEHRHPGLGELGQGPDSYGSPSFRSTPE
APYASLTEIEHLVQSVCKSYRETCQLRLEDLLRQRSNIFSREEVTGYQRKSMWEMWERCA
HHLTEAIQYVVEFAKRLSGFMELCQNDQIVLLKAGAMEVVLVRMCRAYNADNRTVFFEGK
YGGMELFRALGCSELISSIFDFSHSLSALHFSEDEIALYTALVLINAHRPGLQEKRKVEQ
LQYNLELAFHHHLCKTHRQSILAKLPPKGKLRSLCSQHVERLQIFQHLHPIVVQAAFPPL
YKELFSTETESPVGLSK

>hsa:6256
MDTKHFLPLDFSTQVNSSLTSPTGRGSMAAPSLHPSLGPGIGSPGQLHSPISTLSSPING
MGPPFSVISSPMGPHSMSVPTTPTLGFSTGSPQLSSPMNPVSSSEDIKPPLGLNGVLKVP
AHPSGNMASFTKHICAICGDRSSGKHYGVYSCEGCKGFFKRTVRKDLTYTCRDNKDCLID
KRQRNRCQYCRYQKCLAMGMKREAVQEERQRGKDRNENEVESTSSANEDMPVERILEAEL
AVEPKTETYVEANMGLNPSSPNDPVTNICQAADKQLFTLVEWAKRIPHFSELPLDDQVIL
LRAGWNELLIASFSHRSIAVKDGILLATGLHVHRNSAHSAGVGAIFDRVLTELVSKMRDM
QMDKTELGCLRAIVLFNPDSKGLSNPAEVEALREKVYASLEAYCKHKYPEQPGRFAKLLL
RLPALRSIGLKCLEHLFFFKLIGDTPIDTFLMEMLEAPHQMT

```
>hsa:6257
MSWAARPPFLPQRHAAGQCGPVGVRKEMHCGVASRWRRRRPWLDPAAAAAAAVAGGEQQT
PEPEPGEAGRDGMGDSGRDSRSPDSSSPNPLPQGVPPPSPPGPPLPPSTAPSLGGSGAPP
PPPMPPPPLGSPFPVISSSMGSPGLPPPAPPGFSGPVSSPQINSTVSLPGGGSGPPEDVK
PPVLGVRGLHCPPPPGGPGAGKRLCAICGDRSSGKHYGVYSCEGCKGFFKRTIRKDLTYS
CRDNKDCTVDKRQRNRCQYCRYQKCLATGMKREAVQEERQRGKDKDGDGEGAGGAPEEMP
VDRILEAELAVEQKSDQGVEGPGGTGGSGSSPNDPVTNICQAADKQLFTLVEWAKRIPHF
SSLPLDDQVILLRAGWNELLIASFSHRSIDVRDGILLATGLHVHRNSAHSAGVGAIFDRS
LSRVLTELVSKMRDMRMDKTELGCLRAIILFNPDAKGLSNPSEVEVLREKVYASLETYCK
QKYPEQQGRFAKLLLRLPALRSIGLKCLEHLFFFKLIGDTPIDTFLMEMLEAPHQLA

>hsa:7421
MEAMAASTSLPDPGDFDRNVPRICGVCGDRATGFHFNAMTCEGCKGFFRRSMKRKALFTC
PFNGDCRITKDNRRHCQACRLKRCVDIGMMKEFILTDEEVQRKREMILKRKEEEALKDSL
RPKLSEEQQRIIAILLDAHHKTYDPTYSDFCQFRPPVRVNDGGGSHPSRPNSRHTPSFSG
DSSSSCSDHCITSSDMMDSSSFSNLDLSEEDSDDPSVTLELSQLSMLPHLADLVSYSIQK
VIGFAKMIPGFRDLTSEDQIVLLKSSAIEVIMLRSNESFTMDDMSWTCGNQDYKYRVSDV
TKAGHSLELIEPLIKFQVGLKKLNLHEEEHVLLMAICIVSPDRPGVQDAALIEAIQDRLS
NTLQTYIRCRHPPPGSHLLYAKMIQKLADLRSLNEEHSKQYRCLSFQPECSMKLTPLVLE
VFGNEIS

>hsa:8856
MEVRPKESWNHADFVHCEDTESVPGKPSVNADEEVGGPQICRVCGDKATGYHFNVMTCEG
CKGFFRRAMKRNARLRCPFRKGACEITRKTRRQCQACRLRKCLESGMKKEMIMSDEAVEE
RRALIKRKKSERTGTQPLGVQGLTEEQRMMIRELMDAQMKTFDTTFSHFKNFRLPGVLSS
GCELPESLQAPSREEAAKWSQVRKDLCSLKVSLQLRGEDGSVWNYKPPADSGGKEIFSLL
PHMADMSTYMFKGIISFAKVISYFRDLPIEDQISLLKGAAFELCQLRFNTVFNAETGTWE
CGRLSYCLEDTAGGFQQLLLEPMLKFHYMLKKLQLHEEEYVLMQAISLFSPDRPGVLQHR
VVDQLQEQFAITLKSYIECNRPQPAHRFLFLKIMAMLTELRSINAQHTQRLLRIQDIHPF
ATPLMQELFGITGS

>hsa:9970
MASREDELRNCVVCGDQATGYHFNALTCEGCKGFFRRTVSKSIGPTCPFAGSCEVSKTQR
RHCPACRLQKCLDAGMRKDMILSAEALALRRAKQAQRRAQQTPVQLSKEQEELIRTLLGA
HTRHMGTMFEQFVQFRPPAHLFIHHQPLPTLAPVLPLVTHFADINTFMVLQVIKFTKDLP
VFRSLPIEDQISLLKGAAVEICHIVLNTTFCLQTQNFLCGPLRYTIEDGARVGFQVEFLE
LLFHFHGTLRKLQLQEPEYVLLAAMALFSPAPYLTDRPGVTQRDEIDQLQEEMALTLQSY
IKGQQRRPRDRSPGTPWIHWSGKMLGPKIGPGSKGAQWLQ

>hsa:9971
MGSKMNLIEHSHLPTTDEFSFSENLFGVLTEQVAGPLGQNLEVEPYSQYSNVQFPQVQPQ
ISSSSYYSNLGFYPQQPEEWYSPGIYELRRMPAETLYQGETEVAEMPVTKKPRMGASAGR
IKGDELCVVCGDRASGYHYNALTCEGCKGFFRRSITKNAVYKCKNGGNCVMDMYMRRKCQ
ECRLRKCKEMGMLAECMYTGLLTEIQCKSKRLRKNVKQHADQTVNEDSEGRDLRQVTSTT
KSCREKTELTPDQQTLLHFIMDSYNKQRMPQEITNKILKEEFSAEENFLILTEMATNHVQ
VLVEFTKKLPGFQTLDHEDQIALLKGSAVEAMFLRSAEIFNKKLPSGHSDLLEERIRNSG
ISDEYITPMFSFYKSIGELKMTQEEYALLTAIVILSPDRQYIKDREAVEKLQEPLLDVLQ
KLCKIHQPENPQHFACLLGRLTELRTFNHHHAEMLMSWRVNDHKFTPLLCEIWDVQ
```

© 2014 by the authors; licensee MDPI, Basel, Switzerland. This article is an open access article distributed under the terms and conditions of the Creative Commons Attribution license (http://creativecommons.org/licenses/by/3.0/).
